# A Hyper-Viscoelastic Constitutive Model for Polyurea under Uniaxial Compressive Loading

**DOI:** 10.3390/polym8040133

**Published:** 2016-04-12

**Authors:** Yang Bai, Chunmei Liu, Guangyan Huang, Wei Li, Shunshan Feng

**Affiliations:** 1 State Key Laboratory Explosion Science and Technology, Beijing Institute of Technology, Beijing 100081, China; baiyang828@163.com (Y.B.); huanggy@bit.edu.cn (G.H.); Leewei451x@gmail.com (W.L.); 2 First Research Institute of The Ministry of Public Security of PRC, Beijing 100048, China; liuchunmei0127@163.com

**Keywords:** Ogden, K-BKZ, compression, hyperelastic, viscoelastic, polyurea

## Abstract

A hyper-viscoelastic constitutive model for polyurea by separating hyperelastic and viscoelastic behaviors has been put forward. Hyperelasticity represents the rate-independent responses at low strain rates, described by a three-parameter Mooney-Rivlin model and a third Ogden model. By fitting the quasi-static experimental data, the Ogden model is more appropriate to describe the hyperelastic behaviors for its better agreement at strain over 0.3. Meanwhile, viscoelasticity represents the rate-dependent responses at high strain rates, described by the Standard Linear Solids (SLS) model and the K-BKZ model. By fitting the experimental data of split Hopkinson pressure bar (SHPB), the SLS model is more appropriate to describe the viscoelastic behaviors at strain rates below 1600 s^−1^, but the K-BKZ model performs better at strain rates over 2100 s^−1^ because of the substantial increase of Young’s modulus and the state of polyurea transforming from rubbery to glassy. The K-BKZ model is chosen to describe the viscoelastic behavior, for its low Root Mean Square Error (RMSE) at strain rates below 1600 s^−1^. From the discussion above, the hyper-viscoelastic constitutive model is chosen to be the combination of the Ogden model and the K-BKZ model.

## 1. Introduction

Polyurea is an elastomeric material formed by the chemical reaction between an isocyanate and an amine. Due to its good performance in terms of corrosion, abrasion, and impact resistance, polyurea is widely used in various industries, especially in the coating industry. Additionally, because of the development of spray polyurea elastomer technology (SPUA), polyurea can be directly sprayed on (or just adhere to) the surface of structural components to enhance the mechanical strength and durability of civil and military structures in harsh environments [1,2,3].

In the past decades, many researchers have investigated the use of polyurea as a protective coating on military structures to mitigate damage caused by severe threats, such as blast and projectiles (fragments), due to its ability to absorb and dissipate energy and its high toughness-to-density ratio [4,5,6,7,8].

The dynamic and thermal responses of polyurea are very important for analyzing the mechanism of polyurea’s ability to absorb and dissipate energy. However, under severe loading conditions, those responses are difficult to capture by experiments, which may be very expensive and time consuming. Thus, an accurate constitutive model is necessary for numerical analysis to solve this problem.

Previous studies have demonstrated that polyurea shows elastic and almost incompressible behaviors, and its stress-strain behavior depends on strain rate, temperature, and pressure [9,10].

Mooney-Rivlin and Ogden models have been widely used to describe the dynamic response of incompressible rubbery materials [11]. Some researche utilized these two models to describe the dynamic responses of polyurea. For example, Ackland *et al.* [6] applied a two-parameter Mooney-Rivlin model and Xue *et al.* [7,11], a six-term one. Others developed new models based on these two: Gamonpilas *et al.* [12] proposed a non-linear viscoelastic constitutive model for polyurea assuming a separable time- and strain-dependent material behavior, which showed good agreement with experimental results for a wide range of strain rates under both compressive and tensile loadings. Specifically, the strain-dependent function was described by a nine-parameter Mooney-Rivlin model, and the time function was assumed to follow a Prony series. Li *et al.* [13] proposed a hyper-viscoelastic constitutive model for polyurea which incorporated hyperelastic and viscoelastic behavior of polyurea. The Odgen model was used for the hyperelastic part and a special form of the Zapas kernel for the damping function was used to capture the viscoelastic behavior of polyurea.

Meanwhile, some models had been developed specifically for polyurea. Amirkhizi *et al.* [9] proposed an experiment-based nonlinear viscoelastic constitutive model incorporating the classical Williams-Landel-Ferry (WLF) time-temperature transformation and pressure sensitivity, which showed good agreement with the experimental results at high strain rates. Elsayed *et al.* [14] proposed a constitutive model represented by an elastoplastic network and several viscoelastic mechanisms; the elastoplastic component described long-term behavior and permanent damage, while viscoelastic components accounted for time-dependent viscous dissipation.

Although the aforementioned models for polyurea are almost relatively successful in replicating experimental data, some of them are too complicated to be used as a user-defined material subroutine and imported into the commercially available finite element software, such as LS-DYNA and ABAQUS. Another problem is that the state of polyurea transformed with the increase of strain rates [15,16], yet none of the aforementioned models can be appropriately applied to describe the viscoelastic behavior with it at high strain rates. In this paper we take the state of polyurea transformed at high strain rates into consideration, and propose a constitutive model with accurate, but also simple, formulation for polyurea based on our own experiments. The ideas of this paper are organized as follows:

(i) To describe the hyperelastic behavior of polyuea at low strain rates, and choose a more accurate model between three-parameter Mooney-Rivlin model and the third Ogden model based on quasi-static compression experimental data.
(ii) To describe the viscoelastic behavior of polyuea at high strain rates, and choose a more accurate model between the SLS model and the K-BKZ model based on the SHPB experimental data.
(iii) To combine the two models chosen from the previous two steps to yield the constitutive model for polyurea.

The hyper-viscoelastic constitutive model is finally decided as the combination of third Ogden model and the K-BKZ model.

## 2. Materials and Methods

### 2.1. Speicmens Preparation

**Figure 1:**
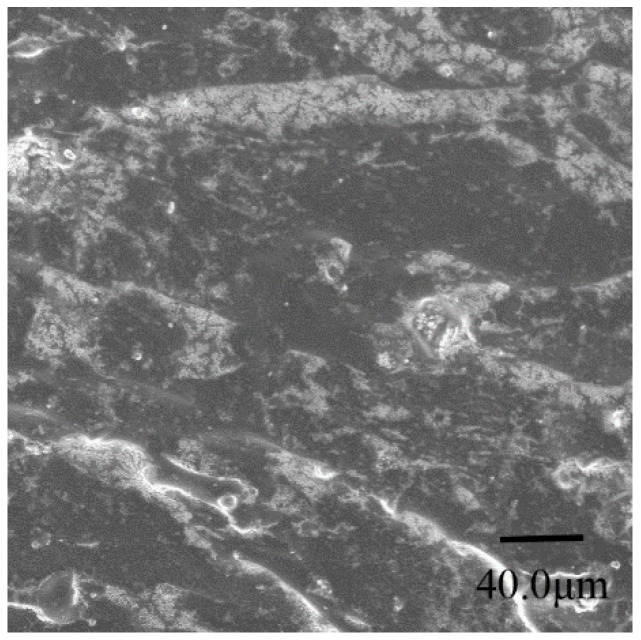
The microstructure of polyurea under SEM.

The polyurea samples used in this study were obtained from the Qingdao Jialian Research and Production Division, Qingdao, China. The polyurea was made from rapid chemical reaction between diphenylmethane diisocynante (MDI, 143L, Wanhua Chemical Group, Yantai, China) and diamine (Unilink 4200, UOP, Des Plaines, IL, USA); the molar ratio of the former to the latter is 1.05. The polyurea was sprayed into sheets to cure at room temperature (RT), after annealing at RT for one month, the experimental specimens were processed from the cast sheets. The density of polyurea is 1.0 ± 0.05 g/cm^3^ and the glass transition temperature (*T*g) is −52.5 °C. The microstructure of polyurea under a scanning electron microscope (SEM) is shown in Figure 1.

The microstructure of polyurea is almost homogeneous and there is no directivity in Figure 1; thus, it can be regarded as a homogeneous and isotropic material.

### 2.2. Experimental

#### 2.2.1. Quasi-Static Experiment

The mechanical behavior of polyurea at low strain rates can be determined by the quasi-static experiment. According to the ASTM D575, the standard specimens for quasi-static compression should be 28.6 ± 0.1 mm in diameter and 12.5 ± 0.1 mm in thickness. In some papers [17,18,19], the compressive specimens were shaped in the form of cylinders with the diameter and height of 10 mm for its convenience to control the compressive displacement and speed. To achieve the maximum true compressive strain of 100%, which corresponds to the engineering compressive strain of 63%, the specimens should be compressed to 3.7 mm at a constant speed. In this paper, the quasi-static compressive specimens are shaped 20 mm in diameter and 10mm in height in this paper, and the strain rate is 10^−3^ s^−1^, 10^−2^ s^−1^ and 10^−1^ s^−1^ corresponding to the compressive speed at 60, 6, and 0.6 mm/min respectively. Specimens were machined using conventional milling procedures from a sheet. Five experiments were performed at each strain rate. An Instron^®^ Model 5969 compression/extension machine (INSTRON, Norwood, MA, USA) was used to perform uniaxial compression test on polyurea.

#### 2.2.2. SHPB Experiment

The split Hopkinson pressure bar (SHPB) is a widely used apparatus to obtain the mechanical behavior at high strain rates (10^2^ s^−1^ ~ 10^4^ s^−1^). The conventional steel SHPB is very useful to test metal materials, but it cannot accurately measure the dynamic responses of soft materials with low strength and low impendence, such as polyurea.

The nominal axial stress in the input and output bar is determined by the equations:

(1) $$\left\{ \begin{array}{l} \sigma_{R}=\frac{1-n}{1+n}\sigma_{I}\\ \sigma_{T}=\frac{4n}{{\left(1+n\right)}^{2}}\sigma_{I} \end{array}\right.$$

where σ_R_ and σ_T_ are the stress amplitude of reflected wave and transmitted wave, respectively; σ_I_ is the stress amplitude of incident pulse; and *n* is the generalized wave impedance ratio: *n* = *A*_s_ρ_s_*c*_s_*/A*_b_ρ_b_*c*_b_, where *A* is cross-sectional area, ρ is density, *c* is wave velocity, and subscripts *s* and *b* represent specimen and incident bar, respectively. The soft materials’ density and wave velocity (*c = (E/*ρ)^1/2^, *E* is Young’s modulus) are smaller than steel, so *n* is so small that most of the incident pulse is reflected into the incident bar with only a small portion of incident pulse transmitted though the specimens into the transmission bar. This means the amplitude of the transmitted strain signal ε_t_ (t) is too small to reach a better signal-to-noise ratio for the measured results in the SHPB signal collecting system. Previous experiments showed that polyurea in this paper has a mass density of 1.0 ± 0.05 g/cm^3^, and Young’s modulus above 100 MPa (strain rate below 900 s^−1^), which has the same results as reference [19].

To achieve a measurable transmitted pulse, numerous methods have been tried. The simplest and most widely applied approach is using more sensitive strain gauges, such as semiconductor strain gauges, instead of traditional resistance strain gauges [11,20]. Another simple approach is using pulse shapers which cannot observably increase the amplitude of the transmission signal, but reduce the strain level by reducing the initial slope of the pulse when the dynamic stress equilibrium is acquired. Sarva *et al.* [16] used copper pulse shapers to help improve dynamic equilibrium and also dampen the high-frequency components in the stress pulses, thus reducing the dispersive effects. Nie *et al.* [21] employed pulse-shaping techniques to create loading histories on the soft specimens so that the specimens deform approximately under dynamic stress equilibrium at constant strain rates. Chen *et al.* [22] developed a modified SHPB technique with a hollow aluminum alloy transmission bar, which can increase the amplitude of the transmitted strain signal and lengthen the rising time of the incident pulse to ensure stress equilibrium and homogeneous deformation in soft specimens. At the same time, non-metallic bars have been proposed and extensively analyzed because of their fewer signal oscillations and higher signal-to-noise ratio, Gray and Blumenthal recommended using low-impedance Hopkinson bars to test polymeric materials [23]. Sawas *et al.* [24] introduced an all-polymeric split Hopkinson bar (APSHB) experiment which could provide both lower signal-to-noise ratio data and a longer input pulse for higher maximum strain. Zhao *et al.* [25] used a viscoelastic SHPB setup to test foam whose ratio of impedance to steel is 0.09%. Doman *et al.* [26] used a Polymeric Split Hopkinson Pressure Bar (PSHPB) supported by high-speed photography.

As noted above, there are four approaches to test the dynamic mechanical behaviors of soft materials, each with their own advantages and disadvantages: using semiconductor strain gauges can observably increase the amplitude of transmission signal, but the large amount of noise cannot be neglected, which makes detailed features difficult to be identified. Using pulse shapers can achieve dynamic stress equilibrium but will result in signal overlap between the incident pulse and reflected pulse. Using the hollow aluminum alloy transmission bar technique can observably decrease the noise than a solid bar, but the calculated strain-rate histories appear significantly noisier than solid bar [27]. Using non-metallic bars can achieve a closer impedance match between soft materials and bars, so higher signal-to-noise ratios can be achieved compared with other approaches. However, wave attenuation and dispersion in viscoelastic bars should be noted, and the mathematical analysis method in viscoelastic bars is more complicated than metallic bars. Another problem that should be noted is that in order to achieve a higher strain rate, a higher impact velocity is needed, which will result in the plastic deformation of the bars. Furthermore, the effect of the dynamic mechanical behavior of viscoelastic bars cannot be neglected, which results from some parameters such as environmental temperatures, moisture levels, and aging factors [28].

Although Jonson *et al.* [27] had compared these four approaches, there was no final conclusion about which approach is the most advantageous. In this paper, we employed the semiconductor strain gauges associated with a solid aluminum alloy bar to test polyurea. Compared with a hollow aluminum alloy bar, solid one can achieve a higher signal-to-noise ratio with a simple structure which is easier to manufacture; additionally, by choosing a longer striker bar and appropriate specimen size, a longer rising time of incident pulse and stress equilibrium and homogeneous deformation in polyurea specimens can be achieved. Compared with non-metallic alloy bars, on the other hand, solid aluminum alloy bars can achieve a higher strain rate with simpler mathematical analysis. Additionally, semiconductor strain gauges can increase the amplitude of the transmission signal, and in order to ensure the integrality of the pulse, pulse shapers were not considered.

The striker bar was propelled forward against the incident pressure bar by the gas produced from a gas cylinder. To achieve a measurable transmitted pulse, the aluminum SHPB was instrumented with semiconductor strain gauges: two 120 Ω semiconductor strain gauges were located on the transmitter bar and two 120 Ω resistor strain gauges were located on the incident bar (semiconductor strain gauges are 50 times more sensitive than resistor strain gauges). The gauges were arranged in a half-bridge configuration to eliminate any effects of bending. In order to achieve longer duration, a 800 mm striker bar was used. Specifications of the aluminum SHPB bars are listed in Table 1.

**Figure 2:**
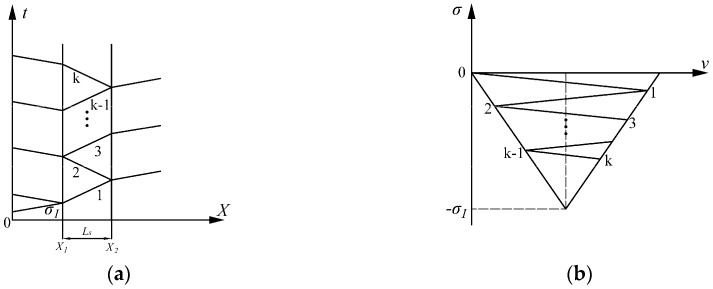
The process propagation of stress waves in the specimen, (**a**) is the *X-t* diagram, showing the process of stress wave propagation between bars and the specimen; and (**b**) is the σ*-v* diagram, showing the value of stress in the specimen changing with k.

The size of specimen plays a vital role in ensuring the validation of the experimental results for soft materials. The optimal length-to-diameter (*L*/*D*) ratio of specimens for the SHPB test is 0.5, which has been widely used to minimize the effects of inertia and friction [27,29]. The length of soft material specimens must be short enough to ensure homogeneous deformation and stress equilibrium during the experiments. The process of stress wave propagation in the specimen is shown in Figure 2.

Where σ_I_ is the same as mentioned in the Equation (1). *X*_1_ is the incident bar-specimen interface, *X*_2_ is the specimen-transmission bar interface, *Ls* is the length of specimen, and k is the times of the incident pulse traveling between *X*_1_ and *X*_2_.

The total stress in the specimen after the incident pulse travels between *X*_1_ and *X*_2_ for *k* times is:

(2) $$\sigma_{k}=\left[1-{\left(\frac{n-1}{n+1}\right)}^{k}\right]\frac{A}{A_{s}}\sigma_{I}$$

The stress difference between *X*_1_ and *X*_2_ is:

(3) $$\Delta \sigma_{k}=\sigma_{k}-\sigma_{k-1}={\left(\frac{n-1}{n+1}\right)}^{k-1}\frac{A}{A_{s}}\sigma_{I}$$

The dimensionless stress difference between *X*_1_ and *X*_2_ is:

(4) $$\alpha_{k}=\frac{\Delta \sigma_{k}}{\sigma_{k}}=\frac{2{\left(n-1\right)}^{k-1}}{{\left(n+1\right)}^{k}-{\left(n-1\right)}^{k}}$$

The specimen is considered to reach dynamic stress equilibrium when the dimensionless stress difference is less than 5% [30].

**Figure 3:**
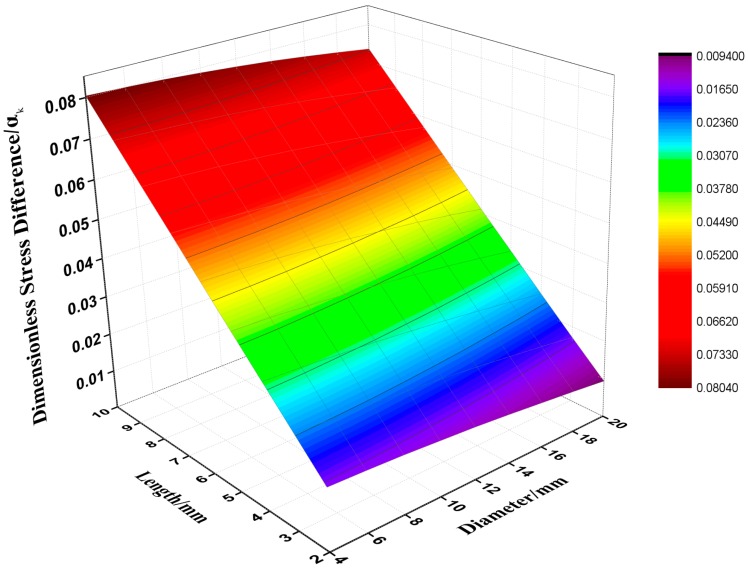
The size of the specimen corresponding to the dimensionless stress difference.

From Figure 3, it can be seen that when the length of specimen is less than 6 mm, the dimensionless stress difference is less than 5%. On the other hand, the thin specimen will make the interfacial friction between the specimen and the bar ends significant. Thus, the length of the specimen is designed to be 5 mm in this paper. Another point that should be taken into consideration is that to achieve higher strain rates, a higher propelling gas pressure is needed, a higher velocity the striker bar will be launched against the incident bar, which may result in permanent deformation of bars and damage to experimental equipment, especially the semiconductor strain gauges. For this reason, gas pressures higher than those used in this paper were not tried. Since the specimens for the SHPB tests are shorter than quasi-static experiments, the cylindrical specimens are easily punched from the supplied sheets which could make the circumferential surface smoother. Five specimens were tested at each strain rate.

The effect of friction between the bars and specimens may lead to localized deformation of the specimen called barreling.In order to minimize the effect, the specimen surfaces which contact the bars were lubricated with a thin layer of petroleum jelly and the contacting bars’ end surfaces were lapped and polished. A Vision Research^®^ Phantom V-710 high-speed camera was used to capture the specimen deformation throughout the test.

**Figure 4:**
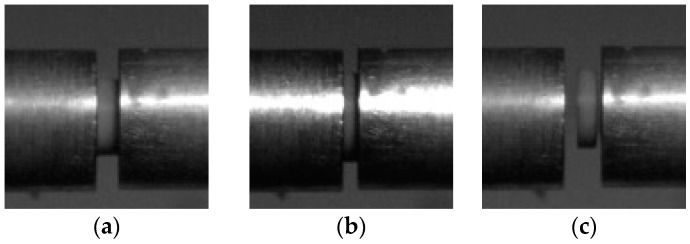
The high-speed photos of deformation of specimen: (**a**) before, (**b**) during, and (**c**) after.

The lateral edges of the specimen remained parallel to the bars in Figure 4b; thus, it can be concluded that the specimen underwent uniform deformation during the test. The lateral edges of the specimen remained almost straight in Figure 4b,c; there was no deformation of barrel found, so the barrelling effect was neglected in this paper.

## 3. Analysis

**Figure 5:**
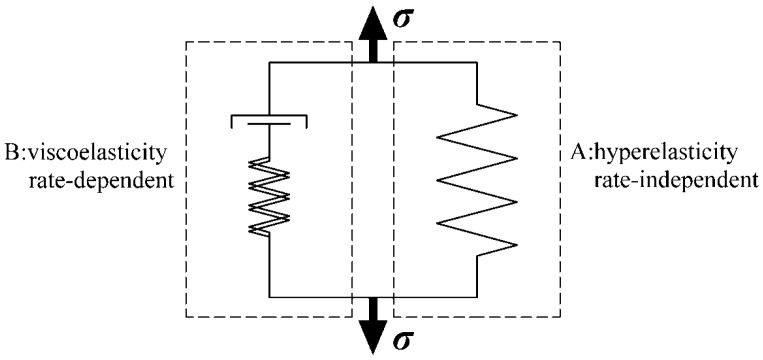
Parallel mechanical response of part A and B.

From the result of the experiments, it can be postulated that the behavior of polyurea combines hyperelasticity and viscoelasticity, which can be described by two parallel parts A and B; the schematic is shown in Figure 5. Part A represents the hyperelasticity which defines the rate-independent response and can be modeled by a nonlinear spring. Part B represents the viscoelasticity which defines the rate-dependent response and can be modeled by a nonlinear spring connected to a nonlinear dashpot.

The total stress is the sum of part A and part B is:

(5) $$\sigma =\sigma_{A}+\sigma_{B}$$

### 3.1. Hyperelasticity

#### 3.1.1. Mooney-Rivlin Theory

According to Mooney and Rivlin [31,32], the constitutive model of isotropic incompressible hyperelastic material can be expressed as:

(6) $$\sigma_{A}=\sigma^{h}=-p_{h}I+\alpha_{1}B+\alpha_{2}B\cdot B$$

where σ_h_ is the Cauchy stress tensor, *p*_h_ is the hydrostatic pressure, α_1_ = 2 (∂W/∂I_1_ + *I*_1_∂W/∂I_2_), α_2_ = −2 ∂W/∂I_2_. *W = W* (*I*_1_*, I*_2_) is a strain energy potential assumed to be a polynomial series. Based on Brown’s analysis [33], three terms have been found in the polynomial series which are sufficient to fit the compressive experimental data [34], *i.e.*:

(7) $$W(I_{1},I_{2})=A_{1}(I_{1}-3)+A_{2}(I_{2}-3)+A_{3}(I_{1}-3)(I_{2}-3)$$

where *A*_1,_ *A*_2_*, A*_3_ are parameters determined by one-dimensional experiments.

For uniaxial loading conditions, the stretch is denoted by λ and the principal stretches are λ_1_ = λ, λ_2_ *=* λ_3_ *=* λ^−1/2^*.* With the definition of the deformation gradient **F** and the left Cauchy-Green deformation tensor **B**, the invariants *I*_1_ = λ^2^ + 2λ^−1^, *I*_2_ = λ^−2^ + 2λ.

Under uniaxial loading, the constitutive model of the hyperelastic part under one-dimensional loading conditions can be written as:

(8) $$\sigma_{A}=\sigma_{11}^{h}=2\lambda (1-\lambda^{-3})\left\{A_{1}\lambda +A_{2}+A_{3}[(\lambda^{2}+2\lambda^{-1}-3)+\lambda (\lambda^{-2}+2\lambda -3)]\right\}$$

where λ = 1 + ε_11_, ε_11_ is the engineering strain in the direction of the uniaxial loading.

#### 3.1.2. Ogden Theory

Another strain energy formulation has been proposed by Ogden [35]:

(9) $$W=\sum_{i=1}^{3}\sum_{j=1}^{n}\frac{u_{j}}{\alpha_{j}}(\lambda_{i}^{*\alpha_{j}}-1)+K(J-1-\ln J)$$

where $\lambda_{i}^{*}$ is a deviatoric principal stretch, $\lambda_{I}^{*}$ = λ_i_*J*^−1/3^; *K* is the bulk modulus; *J* is the Jacobian determinant of the deformation gradient, *J* = λ_1_·λ_2_·λ_3_, and *J* = 1 when the material is incompressible; *u*_j_ and α_j_ are the material parameters and determined by one-dimensional experiments.

Under one-dimensional loading conditions the constitutive model of the hyperelastic part can be written as:

(10) $$\sigma_{A}=\sigma_{11}^{h}=\frac{\partial W}{\partial \lambda }=\sum_{j=1}^{n}u_{j}(\lambda^{\alpha_{j}-1}-\lambda^{-(\alpha_{j}/2+1)})$$

Likewise, λ=1 + ε_11_, *n* is assumed to be 3 in this paper, u_j_, α_j_ (*j* = 1,2,3) are temperature-dependent material parameters, and $\sum_{j\;=\;1}^{n}u_{j}\alpha_{j}\;=\;2\mu$ where μ is the shear modulus; for an incompressible material, μ *= E/(1 +* υ*)*, with *E* representing Young’s modulus at the small strain deformation and υ Poisson’s ratio approximating 0.5.

### 3.2. Viscoelasticity

#### 3.2.1. SLS Theory

**Figure 6:**
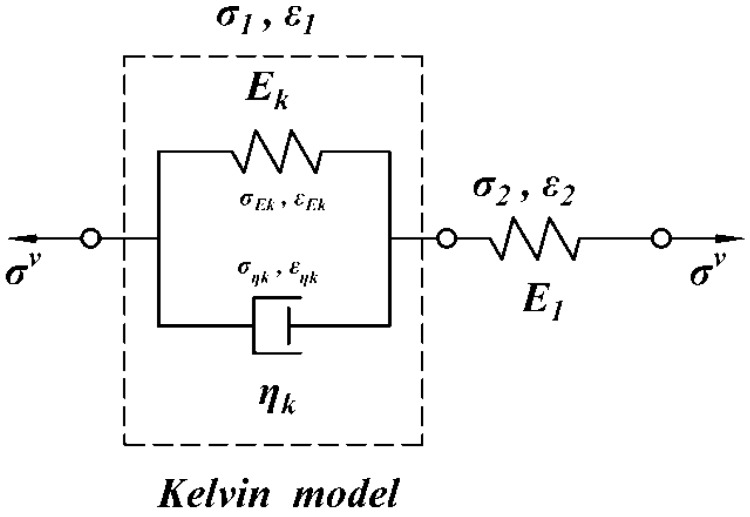
Schematic of the standard linear solid (SLS) model.

A standard linear solid (SLS) model is widely used to describe the viscoelastic material which can be represented by a spring in series with a Kelvin model; the schematic is shown in Figure 6.

The stress and strain of the SLS model are connected as:

(11) $$\begin{array}{l} \sigma^{v}=\sigma_{1}=\sigma_{2}\\ \;\varepsilon =\varepsilon_{1}+\varepsilon_{2} \end{array}$$

Meanwhile the stress and strain of the Kelvin model are connected as:

(12) $$\begin{array}{l} \sigma_{1}=\sigma_{Ek}+\sigma_{\eta k}\\ \varepsilon_{1}=\varepsilon_{Ek}=\varepsilon_{\eta k} \end{array}$$

In the Kelvin model, the spring represents the elastic component of the response, and the relationship of stress and strain represents σ_Ek_ = *E*_k_·ε_Ek_; the dashpot is the viscous component of the response, the relationship of stress and strain is σ_ηk_ = η_k_$\dot{\varepsilon }$_ηk_, where *E*_k_, η_k_ are the material constants.

By the combination of Equation (11) and Equation (12), the constitutive equation of the SLS can be expressed as:

(13) $$(E_{1}+E_{k})\sigma +\eta_{k}\dot{\sigma }=E_{1}E_{k}\varepsilon +E_{1}\eta_{k}\dot{\varepsilon }$$

In Equation(13), *E*_1_, *E*_k_, and η_k_ are the parameters to be obtained, which means the SLS model is a three-parameter model [36]. 

The relaxation modulus *E*(*t*) can be achieved through Laplace transformation:

(14) $$E(t)=\frac{E_{1}}{E_{1}+E_{v}}(E_{v}+E_{1}e^{-\frac{t}{\tau }})$$

where *τ* is the relaxation time τ *=* η_v_/(*E*_1_* + E*_v_), and *t* is the total time.

According to Boltzmann superposition integral, the relationship of stress and strain for the viscoelastic part can be expressed as:

(15) $$\sigma^{v}=\int_{0}^{t}E(t-\xi )\frac{d\varepsilon }{d\xi }d\xi$$

Equation (15) is applicable to finite strain. In order to highlight the undetermined parameters which are obtained by fitting the data of experiments, Equation (15) can be converted to:

(16) $$\sigma_{B}=\sigma^{v}=\int_{0}^{t}\left(A_{4}+A_{5}e^{-\frac{t-\xi }{A_{6}}}\right)\dot{\varepsilon }d\xi$$

where *A*_4_ = *E*_1_*E*_v_/(*E*_1_ + *E*_v_), *A*_5_ = $E_{1}^{2}$/(*E*_1_ + *E*_v_), *A*_6_ = τ *=* η_v_/(*E*_1_* + E*_v_), $\dot{\varepsilon }$ is the true strain rate converted from an engineering strain rate $\dot{\varepsilon }\;$= $\dot{\varepsilon }$_e_/(1 − ε_e_) by the function of the true strain ε=−ln(1 − ε_e_) (ε and ε_e_ are scalars).

#### 3.2.2. K-BKZ Theory

The stress state of viscoelastic materials depends on strain and strain rate histories. Polyurea is regarded as a homogeneous, isotropic, and incompressible material, and the constitutive model for this kind of material can be described by K-BKZ model [37,38,39]:

(17) $$\sigma^{v}=-p^{v}I+F(t)\cdot \overset{t}{\underset{\tau =-\infty }{\Omega }}\left\{C(\tau )\right\}\cdot F^{T}(t)$$

where σ^v^ is the Cauchy stress tensor, *p*^v^ is the pressure for a viscoelastic material, and Ω is a frame-independent matrix function which describes the effect of strain history on stress. Numerous approximations have been proposed to represent the functional Ω [38,40]. Yang *et al.* [34] assumed a simplified form based on the BKZ model:

(18) $$\overset{t}{\underset{\tau =-\infty }{\Omega }}\left\{C(\tau )\right\}=\int_{-\infty }^{t}\phi (I_{1}^{'},I_{2}^{'})m(t-\tau )\dot{E}(\tau )d\tau$$

*φ*($I_{1}^{＇}$, $I_{2}^{＇}$) is the damping function [41,42]:

(19) $$\phi (I_{1}^{'},I_{2}^{'})=\frac{A_{7}}{(A_{7}-3)+A_{8}I_{1}^{'}+(1-A_{8})I_{2}^{'}}$$

where $I_{1}^{＇}$ and $I_{2}^{＇}$ are the first and second invariants of the strain tensor *C*(τ).

The relaxation function *m* (t − τ) is described by an exponential series:

(20) $$m(t-\tau )=\sum_{i=1}^{N}\exp(-\frac{t-\tau }{\theta_{i}})$$

where θ*_i_* is the relaxation time, *N* can be assigned different value, Wagber *et al.* [40] set *N* equal to 8, Osaki *et al.* [43] selected N equal to 5, while other researchers selected *N* to be much smaller: Wang *et al.* [44,45] took 2 as the value of *N*, and Yang *et al.* proposed the value as 1 [34], for the reason that the relaxation time is determined by the loading time or rate. A single relaxation could adequately describe the mechanical response of polymeric materials in the SHPB experiment, and the fitting curves show very good agreement with the experimental data. In order to simplify the constitutive model by decreasing the unknown parameters, in this paper *N* = 1.

The strain rate is defined by:

(21) $$\dot{E}=({\dot{F}}^{T}\cdot F+F^{T}\cdot \dot{F})/2$$

Under uniaxial loading, $I_{1}^{＇}$, $I_{2}^{＇}$ is equal to *I*_1_, *I*_2_ in the hyperelastic part, and the strain rate $\dot{E\;}$= $\dot{\lambda }$_1_λ_1_ = $\dot{\lambda }\lambda$, where $\dot{\lambda }$ is the stretching rate equal to the engineering strain rate $\dot{\varepsilon }$. Meanwhile, it is assumed that the deformation history for *t* < 0 has no effect on the stress at time *t* > 0, and Ω can be expressed as:

(22) $$\overset{t}{\underset{\tau =0}{\Omega }}\left\{C(\tau )\right\}=\int_{0}^{t}\frac{A_{7}}{(A_{7}-3)+A_{8}I_{1}+(1-A_{8})I_{2}}\exp(-\frac{t-\tau }{A_{9}})\lambda \dot{\lambda }d\tau$$

The viscoelastic constitutive model under one-dimensional loading conditions can be written as:

(23) $$\sigma_{11}^{v}=-p^{v}+\lambda^{2}\int_{0}^{t}\frac{A_{7}}{(A_{7}-3)+A_{8}I_{1}+(1-A_{8})I_{2}}\exp(-\frac{t-\tau }{A_{9}})\lambda \dot{\lambda }d\tau$$

The viscoelastic pressure *p*^v^ is obtained from the condition transverse stress $\sigma_{22}^{v}$ = $\sigma_{33}^{v}$ = 0, and $\dot{E}$_22_ = $\dot{\lambda }$_2_λ_2_ = −λ^−^^2^$\dot{\lambda }$/2:

(24) $$p^{v}=-\frac{1}{2}\lambda^{-1}\int_{0}^{t}\frac{A_{7}}{(A_{7}-3)+A_{8}I_{1}+(1-A_{8})I_{2}}\exp(-\frac{t-\tau }{A_{9}})\lambda^{-2}\dot{\lambda }d\tau$$

Substitution of Equation (24) into Equation(23), the constitutive model of the viscoelastic part is:

(25) $$\begin{array}{ll} \sigma_{B}=\sigma_{11}^{v}= & \frac{1}{2}\lambda^{-1}\int_{0}^{t}\frac{A_{7}}{(A_{7}-3)+A_{8}I_{1}+(1-A_{8})I_{2}}\exp(-\frac{t-\tau }{A_{9}})\lambda^{-2}\dot{\lambda }d\tau \\ & +\lambda^{2}\int_{0}^{t}\frac{A_{7}}{(A_{7}-3)+A_{8}I_{1}+(1-A_{8})I_{2}}\exp(-\frac{t-\tau }{A_{9}})\lambda \dot{\lambda }d\tau \end{array}$$

## 4. Results and Discussion

### 4.1. Properties of Polyurea

**Figure 7:**
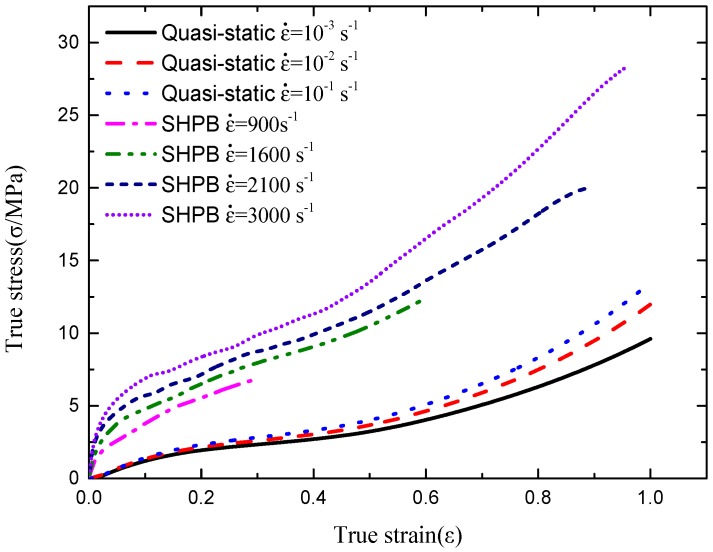
The true stress-strain curves of polyurea at different strain rates.

The average data of five tested specimens in each strain rate was used to draw up true stress-strain true curves. The relationship between true and engineering stress is σ = σ_e_ (1 − ε_e_), where *σ* and σ_e_ are the true and engineering stress respectively. Average true stress-strain curves of five tested specimens in each strain rate are presented in Figure 7.

From Figure 7, similarities can be found that the stress-strain curves in quasi-static experiment at strain rate 10^−3^, 10^−2^, and 10^−1^ s^−1^, especially in the starting stage of the deformation the curves almost overlap. It can be concluded that the mechanical behavior responses of polyurea are rate-independent at low strain rates. Meanwhile the stress-strain curves at strain rate 900, 1600, 2100, and 3000 s^−1^ achieved by the SHPB experiment are different, and more obvious differences can be noticed compared with the quasi-static experimental curves. The stress increased at the same strain as the strain rate increased; thus, it can be concluded that the mechanical behavior responses of polyurea is rate-dependent at high strain rates.

**Figure 8:**
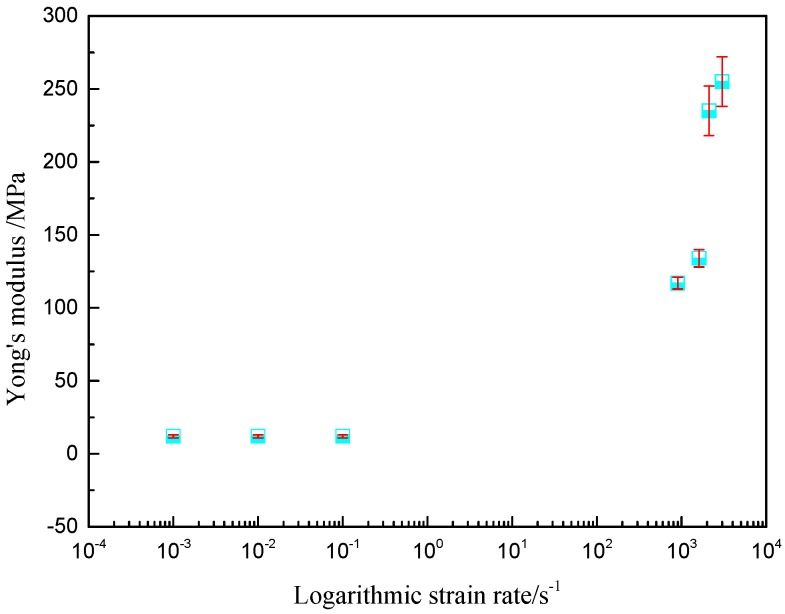
Young’s modulus of polyurea at different logarithmic strain rates.

The Young’s modulus of polyurea at different high strain rates are shown in Figure 8.

**Figure 9:**
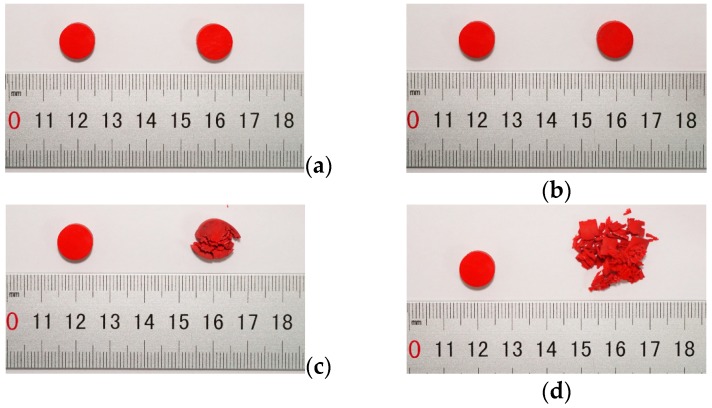
The failure forms of polyurea at different strain rates: (**a**) strain rate 900 s^−1^; (**b**) strain rate 1600 s^−1^; (**c**) strain rate 2100 s^−1^; (**d**) strain rate 3000 s^−1^.

The Young’s modulus of polyurea remains almost constant under quasi-static condition, but when strain rate spanned to high strain rate (above 900 s^−1^) the amplification of Young’s modulus is over 875%. From Figure 8, a clear trend is shown in the diagram, where the Young’s modulus increases with the amplification of strain rate at high strain rates. It can be concluded that the Young’s modulus of polyurea is also sensitive to the strain rate at a high stain rate [46,47]. When the strain rate increased from 900 to 1600 and from 2100 to 3000 s^−1^, the Young’s modulus, respectively, increased by 14.53% and 8.51%, but when the strain rate spanned from 1600 to 2100 s^−1^, the Young’s modulus increased by 75.37%. The failure forms of polyurea changed when the strain rate spanned from 1600 to 2100 s^−1^, as shown in Figure 9; the left and right are the specimens before and after the respective experiments.

From the Figure 9a,b it can be seen that the failure form of polyurea is rubbery deformation, and there is almost no permanent deformation at strain rates 900 and 1600 s^−1^. However, when the strain rates spanned to 2100 s^−1^, the failure form transformed from rubbery deformation to brittle failure, because high strain rates induce a transition from a rubbery to a glassy state [16,48], and with the increase of the strain rate the brittle degree deepens, as shown in the Figure 9c,d. It can be implied that polyurea transforms from a rubber-like to a glass-like material when the strain rate spanned from 1600 to 2100 s^−1^.

### 4.2. Curves Fitting

**Figure 10:**
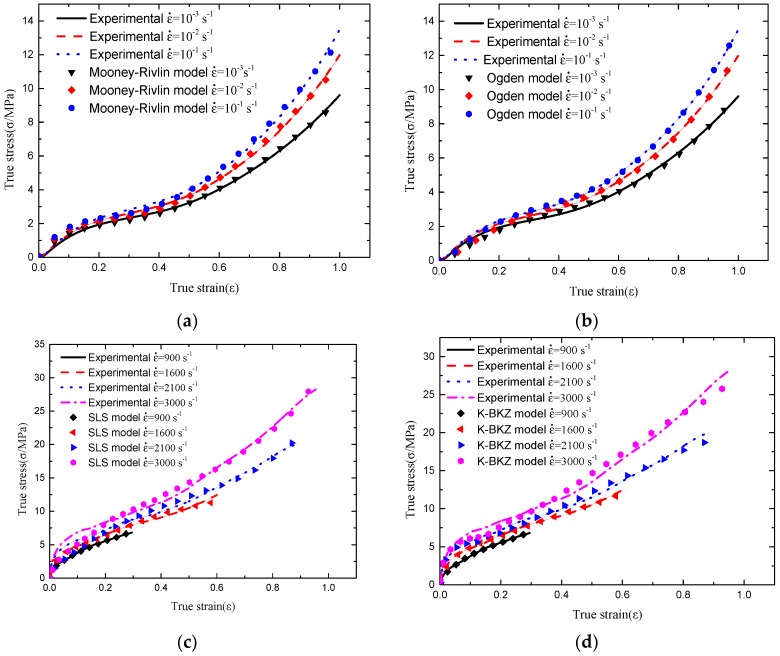
Comparison between experimental data and theoretical model: (**a**) quasi-static compression experimental data and Mooney-Rivlin model; (**b**) quasi-static compression experimental data and Ogden model; (**c**) SHPB experimental data and SLS model; and (**d**) SHPB experimental data and K-BKZ model.

The Mooney-Rivlin model and the Ogden model are used to fit the quasi-static experimental stress-strain curves. Meanwhile, the SLS model and the K-BKZ model are used to fit the SHPB experimental stress-strain curves. The fitting results are shown in Figure 10. The Levenberg-Marquardt algorithm (LM) is implemented to fit the models and experimental data.

The comparison illustrated in Figure 10. a,b shows that the Ogden model performs better agreement with the experimental data than the Mooney-Rivlin model, especially when strain reaches over 0.3 at strain rates of 10^−2^ and 10^−1^ s^−1^. The comparison illustrated in Figure 10. c,d shows that the SLS model performs better agreement with the experimental data than the K-BKZ model at strain rates of 900 and 1600 s^–1^, but at strain rates of 2100 and 3000 s^−1^, the K-BKZ model performs much better than the SLS model, especially in the starting stage of deformation. The Root Mean Square Error (RMSE) is implemented to measure the quality of fitting between theoretical models and the experimental data, and to choose which model is more suitable to describe the hyperelastic behavior at low strain rates and viscoelastic behavior at high strain rates.

(26) $$RMSE=\frac{1}{n}\sum_{i=1}^{n}{\left(\sigma_{i}^{t}-\sigma_{i}^{e}\right)}^{2}$$

where σ^t^ and σ^e^ are the true stress values of the theoretical model and the experimental data, respectively. The RMSE of different model-fitting data at each strain rate is shown in Table 2; it can be seen that the RMSE of the Mooney-Rivlin model are higher than the Ogden model at strain rates of 10^−2^ and 10^−1^ s^−1^ for the reason of that the Mooney-Rivlin model cannot describe the large deformation (strain over 0.3) of polyuria, as well as the third Ogden model. The RMSE of the Mooney-Rivlin model increases with the increase of strain rate and the result of fit diverges with its goodness of fit approaching 0. On the contrary, the RMSE of the Ogden model decreases with the increase of the strain rate, and the result of fit converges with its goodness of fit approaching 1. This indicates that the third Ogden model has a better performance both in the quality and stability of fitting. Thus, it can be concluded that the third Ogden model is more suitable to describe the hyperelastic behavior of polyurea with higher accuracy at low strain rates than the three-parameter Mooney-Rivlin model. The RMSE of the SLS model are lower than the K-BKZ model at strain rates of 900 and 1600 s^−1^, which shows that the SLS model is more appropriate to describe the viscoelastic behavior of polyurea at strain rates below 1600 s^−1^ when polyurea performs with a rubber-like property. However, when the strain rate spans to over 2100 s^−1^, increasing substantially with the increase of the Young’s modulus of polyurea, the RMSE of the SLS model increases by a greater value than that of the K-BKZ model. Since polyurea transforms from a rubber-like to a glass-like material, the Young’s modulus of polyurea substantially increases, yet the SLS model connot describe this property. From Figure 10. c, it can be seen that the initial stage of the SLS model fitting curves at strain rates of 2100 and 3000 s^−1^ are almost overlapping with the strain rate of 1600 s^−1^, meanwhile K-BKZ performs with good agreement to the experimental data at the initial stage. In general, K-BKZ is more appropriate to describe the viscoelastic behavior of polyurea with higher accuracy when it performs with a glass-like property at a strain rate over 2100 s^−1^.

Thus, the hyper-viscoelastic constitutive model for rubber-like polyurea at strain rates below 1600 s^−1^ is the combination of the third Ogden model and the SLS model, which can be written as:

(27) $$\begin{array}{ll} \sigma =\sigma_{A}+\sigma_{B} & \;=\sum_{j=1}^{3}u_{j}(\lambda^{\alpha_{j}-1}-\lambda^{-(\alpha_{j}/2+1)})+\int_{0}^{t}\left(A_{4}+A_{5}e^{-\frac{t-\xi }{A_{6}}}\right)\dot{\varepsilon }d\xi \\ & =\sum_{j=1}^{3}u_{j}({\left(1+\varepsilon_{11}\right)}^{\alpha_{j}-1}-{\left(1+\varepsilon_{11}\right)}^{-(\alpha_{j}/2+1)})+\int_{0}^{t}\left(A_{4}+A_{5}e^{-\frac{t-\xi }{A_{6}}}\right)\frac{{\dot{\varepsilon }}_{11}}{\left(1-\varepsilon_{11}\right)}d\xi \end{array}$$

Meanwhile the hyper-viscoelastic constitutive model for glass-like polyurea at strain rates over 2100 s^−1^ is the combination of the third Ogden model and the K-BKZ model, which can be written as:

(28) $$\begin{array}{ll} \sigma =\sigma_{A}+\sigma_{B}= & \sum_{j=1}^{3}u_{j}(\lambda^{\alpha_{j}-1}-\lambda^{-(\alpha_{j}/2+1)})\\ & +\frac{1}{2}\lambda^{-1}\int_{0}^{t}\frac{A_{7}}{(A_{7}-3)+A_{8}I_{1}+(1-A_{8})I_{2}}\exp(-\frac{t-\tau }{A_{9}})\lambda^{-2}\dot{\lambda }d\tau \\ & +\lambda^{2}\int_{0}^{t}\frac{A_{7}}{(A_{7}-3)+A_{8}I_{1}+(1-A_{8})I_{2}}\exp(-\frac{t-\tau }{A_{9}})\lambda \dot{\lambda }d\tau \end{array}$$

In Equation(27), ε_11_ is the engineering strain, and λ = 1 + ε_11_ in Equation (28).

The RMSE of the K-BKZ model are low enough at strain rates of 900 and 1600 s^−1^, shown in Table 2. Therefore, the hyper-viscoelastic constitutive model for polyurea is finally decided to be the combination of the third Ogden model and the K-BKZ model at a wide range of strain rates, shown as Equation (28).

## 5. Conclusions

A hyper-viscoelastic constitutive model was presented for polyurea under uniaxial compressive loading by separating hyperelastic and viscoelastic mechanical behaviors in this paper. Hyperelasticity represents the part of rate-independent response at low strain rates, and viscoelasticity represents the part of rate-dependent response at high strain rates. A three-parameter Mooney-Rivlin model and a third Ogden model were used to describe the hyperelastic behavior and fit curves using the quasi-static experimental data. Meanwhile, the SLS model and the K-BKZ model were used to describe the viscoelastic behavior and fit curves using the SHPB data. RMSE was implemented to measure the quality of fitting curves. By comparing the RMSE of fitting curves of the quasi-static experimental data, the third Ogden model was found to be more appropriate to describe the hyperelastic behavior of polyurea than the three-parameter Mooney-Rilvin, because it had better performance both in the quality and stability of fitting, especially at large deformations of strain rates of 10^−2^ and 10^−1^ s^−1^. By comparing the RMSE of fitting curves of the SHPB experimental data, the SLS model was more appropriate to describe the viscoelastic behavior than the K-BKZ model when polyurea performed as a rubber-like property at strain rate below 1600 s^−1^, but when the strain rates spanned to over 2100 s^−1^, the K-BKZ model was more appropriate, because polyurea transformed from a rubber-like to a glass-like material, and the Young’s modulus of polyurea substantially increased. The SLS model could not describe this properties while the K-BKZ model performed in good agreement with the initial stage of the experimental data. Considering the fact that the RMSE of the K-BKZ model were low enough at strain rates of 900 and 1600 s^−1^, the hyper-viscoelastic constitutive model for polyurea was finally described by the combination of the third Ogden model and the K-BKZ model at a wide range of strain rates.

## Figures and Tables

**Table 1:** The specifications of the SHPB system.

Parameters	Striker Bar	Incident Bar	Transmission Bar
Length *L* (mm)	800	2,000	2,000
Diameter Φ (mm)	37	37	37
Mass density ρ (kg/m^3^)	2,810		
Young’s modulus (MPa)	70,250		
Strain gauge location *Lg* (mm) (distance from gauge to specimen)	-	1,100	1,100

**Table 2:** The RMSE of different model-fitting data.

Stain Rate (s^−1^)	RMSE	
	Mooney-Rivlin Model	Ogden Model
10^−3^	0.0166	0.0305
10^−2^	0.0371	0.0217
10^−1^	0.0573	0.0118
	**SLS Model**	**K-BKZ Model**
900	0.0033	0.0077
1600	0.0253	0.0816
2100	0.7501	0.1783
3000	0.9457	0.5687
